# *PELP1* inhibition by SMIP34 reduces endometrial cancer progression via attenuation of ribosomal biogenesis

**DOI:** 10.1002/1878-0261.13539

**Published:** 2023-11-01

**Authors:** Xue Yang, Zexuan Liu, Weiwei Tang, Uday P. Pratap, Alexia B. Collier, Kristin A. Altwegg, Rahul Gopalam, Xiaonan Li, Yaxia Yuan, Daohong Zhou, Zhao Lai, Yidong Chen, Gangadhara R. Sareddy, Philip T. Valente, Edward R. Kost, Suryavathi Viswanadhapalli, Ratna K. Vadlamudi

**Affiliations:** ^1^ Department of Obstetrics and Gynecology University of Texas Health San Antonio TX USA; ^2^ Department of Obstetrics and Gynecology, Second Xiangya Hospital Central South University Changsha China; ^3^ Department of Oncology, Xiangya Hospital Central South University Changsha China; ^4^ Department of Obstetrics and Gynecology, Affiliated Hospital of Integrated Traditional Chinese and Western Medicine Nanjing University of Chinese Medicine China; ^5^ Mays Cancer Center University of Texas Health San Antonio TX USA; ^6^ Department of Biochemistry & Structural Biology University of Texas Health San Antonio TX USA; ^7^ Department of Molecular Medicine, Department of Population Sciences, and Greehey Children's Cancer Research Institute University of Texas Health San Antonio San Antonio TX USA; ^8^ Department of Pathology University of Texas Health San Antonio TX USA; ^9^ Audie L. Murphy Division South Texas Veterans Health Care System San Antonio TX USA

**Keywords:** endometrial cancer, mTOR, PELP1, rapamycin, ribosomal biogenesis

## Abstract

Endometrial carcinoma (ECa) is the fourth most common cancer among women. The oncogene *PELP1* is frequently overexpressed in a variety of cancers, including ECa. We recently generated SMIP34, a small‐molecule inhibitor of *PELP1* that suppresses *PELP1* oncogenic signaling. In this study, we assessed the effectiveness of SMIP34 in treating ECa. Treatment of established and primary patient‐derived ECa cells with SMIP34 resulted in a significant reduction of cell viability, colony formation ability, and induction of apoptosis. RNA‐seq analyses showed that SMIP34‐regulated genes were negatively correlated with ribosome biogenesis and eukaryotic translation pathways. Mechanistic studies showed that the Rix complex, which is essential for ribosomal biogenesis, is disrupted upon SMIP34 binding to *PELP1*. Biochemical assays confirmed that SMIP34 reduced ribosomal biogenesis and new protein synthesis. Further, SMIP34 enhanced the efficacy of mTOR inhibitors in reducing viability of ECa cells. SMIP34 is also effective in reducing cell viability in ECa organoids *in vitro* and explants *ex vivo*. Importantly, SMIP34 treatment resulted in a significant reduction of the growth of ECa xenografts. Collectively, these findings underscore the potential of SMIP34 in treating ECa.

## Graphical abstract

The authors show that PELP1 is overexpressed in endometrial cancer. Endometrial cancer cell survival and growth were diminished by PELP1 knockdown or by its inhibition with SMIP34. Mechanistic investigations revealed that SMIP34's binding to PELP1 disrupts the Rix complex, which is necessary for ribosomal biogenesis. PELP1 inhibition with SMIP34 is a unique therapeutic approach for the management of endometrial cancer.

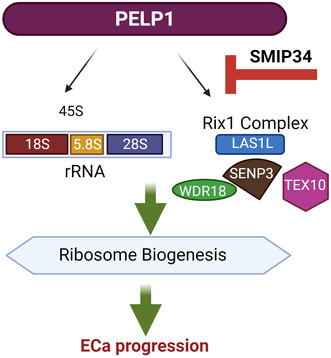

**Abbreviations**
AKT: protein kinase B
ATCC: American Type Culture Collection
DepMap: dependency map
E2: 17‐beta‐estradiol
ECa: endometrial cancer
EEC: endometrioid endometrial cancer
GSEA: gene set enrichment analysis
IHC: immunohistochemistry
IP: immunoprecipitation
mTOR: mammalian target of rapamycin
OPP: *O*‐propargyl‐puromycin
PDE: patient‐derived explant
PDO: patient‐derived organoid
PELP1: proline‐, glutamic acid‐, and leucine‐rich protein 1
SCID: severe combined immunodeficiency
shRNA: short hairpin RNA
TCGA: The Cancer Genome Atlas
TMA: tissue microarray

## 1 Introduction

In the United States, endometrial carcinoma (ECa) is the fourth most common cancer in women and sixth most common malignancy among women to cause death [1]. The endometrioid ECa (EEC), a subtype of ECa, which makes up about 80% of all ECa, is regulated by signaling from estrogen (E2, 17‐β‐estradiol) [2]. Major risk factors for EEC are the imbalance of E2 and progesterone exposures and the use of unopposed E2 therapy [3]. Although therapy with progestins has shown encouraging results for patients receiving uterine‐sparing therapy, adjuvant hormone therapy only offers modest benefits to patients with ECa, and the recurrence rate is around 50% [4]. There is a definite need to identify new treatment targets for ECa.

Despite demonstrating moderate efficacy in clinical studies, targeted drugs including Bevacizumab, and Metformin did not provide lasting remission in individuals with advanced or recurrent ECa [5, 6, 7]. Molecular profiling studies using ECa tissues identified that PTEN, PIK3CA, PIK3R1, and AKT are frequently mutated or amplified. These mutations cause elevated PI3K/AKT/mTOR signaling in type 1 and type 2 ECa and are linked to disease progression and reduced survival [8, 9, 10]. The PI3K/AKT/mTOR pathway is inhibited by several drugs, including mTOR inhibitors; however, they have only a little efficacy in ECa when used as monotherapy [11, 12, 13, 14]. There is an unmet need for the development of novel targeted therapeutics to support current ECa‐directed treatments.

Proline‐, glutamic acid‐, and leucine‐rich protein 1 (*PELP1*) is an oncogene commonly overexpressed in several cancers [15, 16, 17, 18]. PELP1 interacts with mutant p53, regulates its recruitment, and alters epigenetic marks at target gene promoters [19]. PELP1 plays an essential role in several pathways including hormone signaling, mTOR signaling, cell cycle progression, ribosomal biogenesis, and the DNA damage response [20, 21, 22]. Additionally, PELP1 interacts with a variety of chromatin‐modifying complexes, including KDM1A [23], HDAC [24], PRMT [25], CARM1 [26], and contains a histone binding domain [23] that allows it to identify histone modifications. Recent studies identified a small molecule inhibitor SMIP34 that binds and degrades PELP1 [27]; however, its utility in treating ECa remains unknown.

Here, we used EEC cell lines, organoids, tumor tissues, and xenograft animal models to examine the impact of blocking PELP1 oncogenic signaling on the progression of the EEC subtype of ECa. Our results showed that PELP1 suppression caused by PELP1 knockdown or SMIP34 treatment decreased EEC cell viability and enhanced apoptosis. Mechanistic studies revealed that PELP1 inhibition decreased the ribosomal biogenesis and enhanced activation of the apoptosis pathways.

## 2 Materials and methods

### 2.1 Cell lines and reagents

HEC‐1‐A (RRID:CVCL_0293), AN3 CA (RRID:CVCL_0028), RL95‐2 (RRID:CVCL_0505), and HEK293T (RRID:CVCL_0063) cells were purchased from the American Type Culture Collection (ATCC, Manassas, VA, USA) and cultured using ATCC recommended media. Ishikawa cells (RRID:CVCL_2529) were purchased from Sigma (Millipore Sigma, St. Louis, MO, USA). All model cells were free of mycoplasma contamination, and identity was confirmed using short tandem repeat polymorphism analysis (STR). All these four cell lines (HEC‐1‐A, AN3 CA, RL95‐2, and Ishikawa) belong to EEC subtype of ECa. Two PELP1 antibodies were used in this study: (A300‐180A) was purchased from Bethyl Laboratories Inc. (Montgomery, TX, USA); (Abx026482) was purchased from Abbexa LLC (Sugar Land, TX, USA). The p‐Akt (S473), Akt, p‐mTOR (S2448), mTOR, p‐S6 (S235/236), S6, p‐ERK (Thr202/Tyr204), ERK, p‐4E‐BP1 (Thr37/46), 4E‐BP1 and GAPDH antibodies were obtained from Cell Signaling Technology (Beverly, MA, USA). The β‐Actin (A‐2066) and Vinculin antibodies (V9264), were purchased from Millipore Sigma (Burlington, MA, USA). The Ki67 antibody (ab1667) was purchased from Abcam (Cambridge, MA, USA). The WDR18 (15165‐1‐AP), TEX10 (17372‐1‐AP), SENP3 (17659‐1‐AP), and LAS1L (16010‐1‐AP) antibodies were obtained from Proteintech (Rosemont, IL, USA). The WDR18 (HPA050200) antibody used in immunoprecipitation (IP) assay was purchased from Sigma (Millipore Sigma, St. Louis, MO, USA). The synthesis of PELP1 inhibitor SMIP34 was described in earliar publication [27]. mTOR inhibitors rapamycin and AZD8055 were purchased from MedChemexpress (Monmouth Junction, NJ)

### 2.2 Primary ECa cells

Primary ECa cells were generated using patient‐derived ECa tissues collected from the Ob‐Gyn Tissue Core. These tissues were obtained utilizing a procedure authorized by the Institutional Review Board (IRB) of the University of Texas Health San Antonio (UTHSA). The IRB‐approved protocol (project number HSC20190695N) was used to collect the tissue for the study between 2019 and 2023 by UTHSA Tissue Core. All the participants in the study provided written informed consent. These samples were de‐identified, and neither the principal investigator nor the study team had access to clinical linkers or codes. The Declaration of Helsinki and the guidelines established by the UTHSA IRB were followed in all procedures involving human tissues.

### 2.3 Generation of model cell lines

Human specific lentiviral *PELP1*‐shRNA1 (TRCN0000159883; Sigma) and *PELP1*‐shRNA2 (TRCN0000159673; Sigma) particles were used to establish PELP1 knockdown cells. Control cells were generated using lentiviral particles (SHC016‐1EA; Sigma) expressing non‐targeted shRNA. HEK293T and RL95‐2 cells stably expressing PELP1‐GFP were generated using pCDH‐EF1‐GFP‐PELP1 vector. Puromycin (1 μg·mL^−1^) selection was used to produce stable clones, and pooled clones were utilized for all investigations.

### 2.4 Cell viability, clonogenic, and apoptosis assays

The impact of SMIP34 treatment on the cell viability of ECa cells was evaluated using the MTT cell viability assay as described [27]. For colony formation assays, established ECa cells (500 cells/well) and primary ECa cells (2000 cells/well) were plated in 6‐well plates in triplicate and treated with vehicle (control) or SMIP34 for 7 days. Colonies that included at least 50 cells were counted after 2 weeks. The Annexin V/PI kit (BioLegend, San Diego, CA, USA) was used to conduct the analysis of SMIP34's impact on apoptosis as described [27].

### 2.5 RNA‐seq, RT‐qPCR, and bioinformatic analyses

HEC‐1‐A cells were treated with either vehicle (control) or SMIP34 for 24 h, and total RNA was isolated using RNeasy mini kit (Qiagen, Valencia, CA, USA). The Genome Sequencing Facility (UTHSA) established protocol was used to perform RNA‐seq. tophat2 aligner (http://ccb.jhu.edu/software/tophat/index.shtml) was used to map the sequence reads to the UCSC hg19 genome, and HTSeq was used to quantify the reads to the NCBI RefSeq genes. When assessing functional enrichment pathways, significant genes with threshold value of Log_2_FC > 0.5 and adjusted *P* value < 0.05 were employed in the differential expression analysis carried out using deseq2. Pathways were identified using gene set enrichment analysis (GSEA; http://www.broadinstitute.org/gsea/index.jsp) [28]. GSEA analyses were performed using an over‐representation‐based method. The heatmaps of differential genes were produced using R software (version 4.2.1, R Core Team, 2022) and pheatmap package (version 1.0.12, https://cran.r-project.org/web/packages/pheatmap/index.html; Raivo Kolde, 2019). RNA‐seq was deposited in the GEO database under the accession number GSE226850. To verify the selected genes, quantitative real‐time PCR (RT‐qPCR) was performed using gene‐specific primers. The primer sequences are listed in Table S1. The RT‐qPCR method was used as previously described [29]. The *PELP1* gene expression in normal and ECa tissues was analyzed using TNMplot database analysis tool (https://tnmplot.com/) [30]. The Cancer Dependency Map (DepMap) (https://depmap.org/portal/) was used to confirm the essential nature of PELP1 in cancer cells [31]. A PELP1 gene expression correlation study was conducted in ECa by timer2.0 (http://timer.cistrome.org/) [32].

### 2.6 Western blotting, immunoprecipitation, and reporter gene assays

RIPA buffer was used to generate whole cell lysates, and Western blotting analysis was carried out using indicated antibodies as previously mentioned [29]. For IP analysis, cells were lysed using high salt lysis buffer (500 mm NaCl, 50 mm HEPES pH 7.5, 5 mm MgCl_2_, 5% Glycerol, 0.5% NP‐40, EDTA‐free protease inhibitor, 1.75 × 10^4^ U·mL^−1^ Benzonase). Following a 6 h incubation with GFP‐Trap beads (ChromoTek, Rosemont, IL, USA), the lysates were incubated with the designated antibody for 2 h at room temperature. Using the designated antibodies, Western blotting was used to investigate interactions. Using the TurboFect transfection reagent (Thermo Fisher Scientific, Waltham, MA, USA), we transiently transfected ECa cells with reporter plasmids. Renilla luciferase reporter (10 ng) and pHrD‐IRES‐Luc reporter (1 μg) were co‐transfected into ECa cells and after 48 h, cells were treated with SMIP34 (15 μm, 16 h). Using a dual luciferase assay kit (Promega, Madison, WI, USA), the luciferase activity was determined.

### 2.7 Ribosomal biogenesis and global protein synthesis analyses

For ribosomal biogenesis assay, ECa cells were plated in 8‐well chamber slides, treated with SMIP34 (10, 15, 20 μm) for 16 h, then fixed with 100% cold methanol for 15 min and permeabilized with 0.1% Tween‐20 in PBS for 20 min at room temperature. After 1 h blocking with 3% BSA in PBS at room temperature, cells were incubated with mouse anti‐ribosomal protein S6 (rpS6, 1 : 100, Cat# sc‐74459; Santa Cruz, Dallas, TX, USA) for 1 h at room temperature. Cells were then labeled with Alexa 488‐conjugated goat anti‐mouse for 1 h in dark at room temperature (1 : 1000; Thermo Fisher Scientific, Waltham, MA, USA), and visualized using confocal microscopy. Cayman's Protein Synthesis Assay Kit was used for measuring protein synthesis as per the manufacturer protocol (Cat# 601100; Cayman Chemical, Ann Arbor, MI, USA). Briefly, RL95‐2 cells were plated in 8‐well chamber slides and were treated with SMIP34 (15 μm) for 16 h and then treated with cell‐permeable, alkyne‐containing, puromycin analog *O*‐propargyl‐puromycin (OPP, 2.5 μL·mL^−1^) for 90 min at 37 °C. The cells were processed for detection of protein synthesis using 5‐FAM fluorescence and confocal microscopy as per kit protocol. Five fields of each condition were captured, and the mean fluorescence intensity of rpS6 and OPP was calculated by dividing the total fluorescence intensity using imagej (Java 1.8.0, NIH, Bethesda, MD, USA) by the number of cells in each field. Results were normalized to the control group. For measuring global protein synthesis, ECa cells were treated with SMIP34 (5, 10, 15 μm) for 16 h and then treated with puromycin (P7255, Millipore Sigma, St. Louis, MO, USA; 1 μm) for 30 min. Total lysates were analyzed by Western blotting using Anti‐Puromycin [3RH11] antibody (Cat# EQ0001; Kerafast, Boston, MA, USA).

### 2.8 Alignment and visualization of WDR18/PELP1 Rix complex and PELP1/SMIP34 model

The Cryo‐EM structure of WDR18/PELP1 Rix1 complex was retrieved from PDB DataBank with PDB entry 7UWF [33]. The model of SMIP34 binding to full‐length PELP1 was from our previous study [27]. pymol (https://pymol.org/2/) was used to perform 3D structural alignment and visualization.

### 2.9 Patient‐derived explant and organoid studies

Excised tissue samples were processed and cultured *ex vivo* for patient‐derived explant (PDE) studies as previously explained [29] and tissues used were same as described in Section 2.2. The tumor tissue characteristics are listed in Table S2. Patient‐derived organoids (PDO) were developed from de‐identified ECa tumor tissues and cultured in accordance with the ATCC culture guidelines (https://www.atcc.org/en/Guides.aspx). Following the manufacturer's instructions, the Promega^®^ CellTiter‐Glo^®^ 3D‐Superior Cell Viability Assay reagent (Promega, Madison, WI, USA) was used to measure cell viability after 7 days of treatment. A GloMax^®^ Discover System was used to measure the luminescence's intensity (Promega, Madison, WI, USA).

### 2.10 Tissue microarray, immunohistochemistry, and TUNEL analyses

For tissue microarray (TMA) study, EEC TMA from the Ob‐Gyn Tissue Core at UTHSA was used. TMA contained both EEC (*n* = 65) and normal endometrial tissues (*n* = 29). Immunohistochemistry (IHC) analysis was carried out as previously described [27]. The *In Situ* Cell Death Detection Kit (Roche, Indianapolis, IN, USA) was used for TUNEL assay. The percentage of Ki67‐positive and TUNEL‐positive cells in five randomly chosen microscopic fields at 20× magnification was used to construct the proliferative and apoptosis index, respectively. Five randomly chosen microscopic fields and the imagej analysis program was used to quantify the PELP1 staining on xenograft tumor slides (NIH, Bethesda, MD, USA).

### 2.11 *In vivo* xenograft models

All animal experiments were performed after receiving institutional (South Texas Veterans Health Care System, San Antonio, TX, USA) IACUC approval (ACORP 1905‐010). NCI SCID/NCr mice (NOD.CB17‐Prkdcscid/NCrCrl) were purchased from Charles River (Wilmington, MA, USA). Mice were kept in a pathogen‐free environment with a 12/12 h light/dark cycle, temperature: 23 °C, full access to water, and a diet of standard laboratory chow. HEC‐1‐A cells (1 × 10^6^) were mixed with an equal volume of growth factor‐reduced Matrigel and injected subcutaneously into 8‐week‐old female mice. After establishment of tumors, mice were randomly assigned to the control and treatment groups (*n* = 8 tumors per group). SMIP34 (20 mg·kg^−1^·day^−1^ per i.p.) was given to the treatment group whereas vehicle (0.3% hydroxypropyl cellulose) was given to the control group. The volume of the tumor was determined using a modified ellipsoidal formula: tumor volume = 1/2 (*L* × *W* ^2^), where *L* is the longitudinal diameter and *W* is the transverse diameter. Tumor growth was measured with a digital caliper at intervals of 3–5 days. After the experiment was complete, the mice were euthanized, and the tumors were removed, weighed, and prepared for histological analysis.

### 2.12 Statistical analyses

graphpad prism software (version 9.4.1, Boston, MA, USA) was used to examine statistical differences between groups using the unpaired Student's *t*‐test, two‐way and one‐way ANOVA. The means and standard errors of all the data plotted are displayed. Statistical significance was defined as *P* < 0.05.

## 3 Results

### 3.1 PELP1 is overexpressed in ECa and PELP1 knockdown decreased the proliferation of ECa cells

**Fig. 1:**
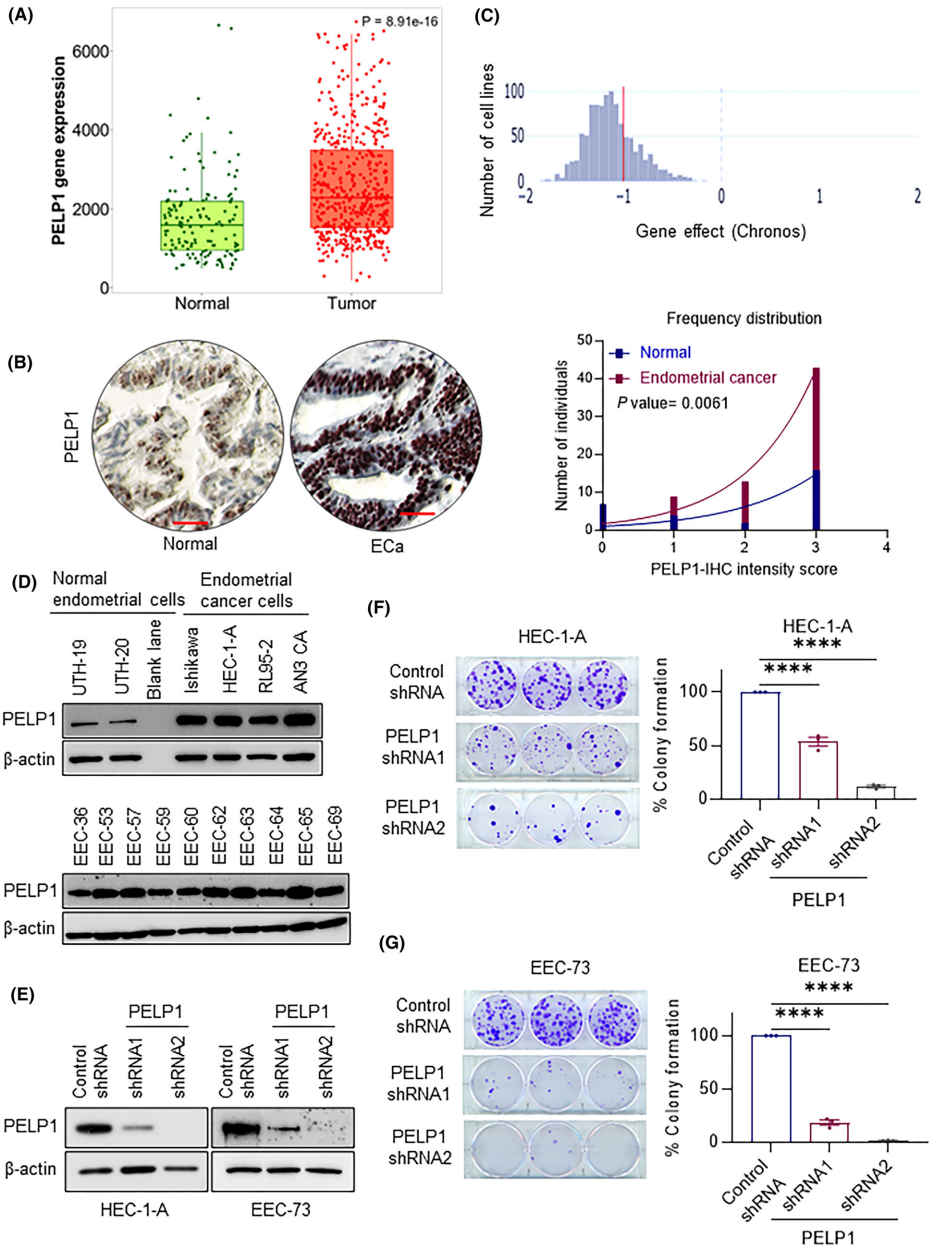
PELP1 expression is upregulated in endometrial cancer (ECa) and PELP1 knockdown decreased the growth of ECa cells. (A) Boxplots of PELP1 expression in normal (*n* = 133) and tumor (*n* = 374) gene array data. Data was obtained from TNMplot database. (B) The expression of PELP1 in tissue samples from patients with EEC (*n* = 65) and from people with normal endometrial tissue (*n* = 29) was assessed, and representative IHC images with PELP1 intensity were displayed, scale bar 100 μm. Frequency distribution of IHC scores is shown in the right panel. Blue and red lines represent frequency of distribution for normal and endometrial cancer respectively. (C) Chrono dependency score of PELP1 in cancer cells using DepMap database. A score of 0 indicates a gene is not essential; correspondingly −1 is comparable to the median of all pan‐essential genes (red line). (D) PELP1 expression in normal endometrial cells and ECa cells was analyzed by Western blotting. The results shown are from one of three experiments (*n* = 3), all of which had similar results. (E) Expression of PELP1 in PELP1 shRNA‐expressing HEC‐1‐A and EEC‐73 cells was analyzed by Western blotting. The results shown are from one of three experiments (*n* = 3), all of which had similar results. Colony formation assays were used to assess the impact of PELP1 knockdown on the growth of HEC‐1‐A (F) and EEC‐73 (G) cells. Results show mean ± SEM, *n* = 3. *P* values are calculated using one‐way ANOVA, ****P < 0.0001.

Through the use of the TNMplot analytic platform, which permits comparison of gene expression between tumor and normal tissues using established databases [30], we looked at whether PELP1 is overexpressed in ECa. Results revealed that ECa had higher PELP1 expression levels than normal tissues (Fig. 1A). Then, using TMA that included 29 normal and 65 ECa specimens, we investigated the expression of PELP1 immunohistochemically. Figure 1B depicts the representative staining in both normal and ECa tissues. Quantitation of IHC staining showed that PELP1 expression is significantly higher in ECa tumor tissues compared to normal tissues (Fig. 1B). We explored the genome‐wide CRISPR/Cas9 proliferation screening database to determine whether PELP1 operates as a crucial gene in cancer cell survival. In this database, gene dependency scores for each gene were determined by Chrono dependency score. Analysis of the DepMap database's data showed that PELP1 is necessary for the survival of cancer cells (Fig. 1C). Further, Western blotting analyses confirmed higher expression of PELP1 in established and primary ECa cell lines compared to normal endometrial cells (Fig. 1D). To examine the functional significance of PELP1 in ECa, we knockdown PELP1 using two different shRNAs (Fig. 1E) and determined whether PELP1 knockdown affected cell survival of ECa model cells (HEC‐1‐A, and EEC‐73). PELP1 knockdown significantly reduced the clonogenic survival of both established HEC‐1‐A and primary EEC‐73 ECa model cells (Fig. 1F,G). Collectively, these results imply that PELP1 is expressed at a high level in ECa relative to normal tissues and that PELP1 is essential for the survival of ECa cells.

### 3.2 SMIP34, a PELP1 inhibitor decreased cell viability and increased apoptosis of ECa cells

**Fig. 2:**
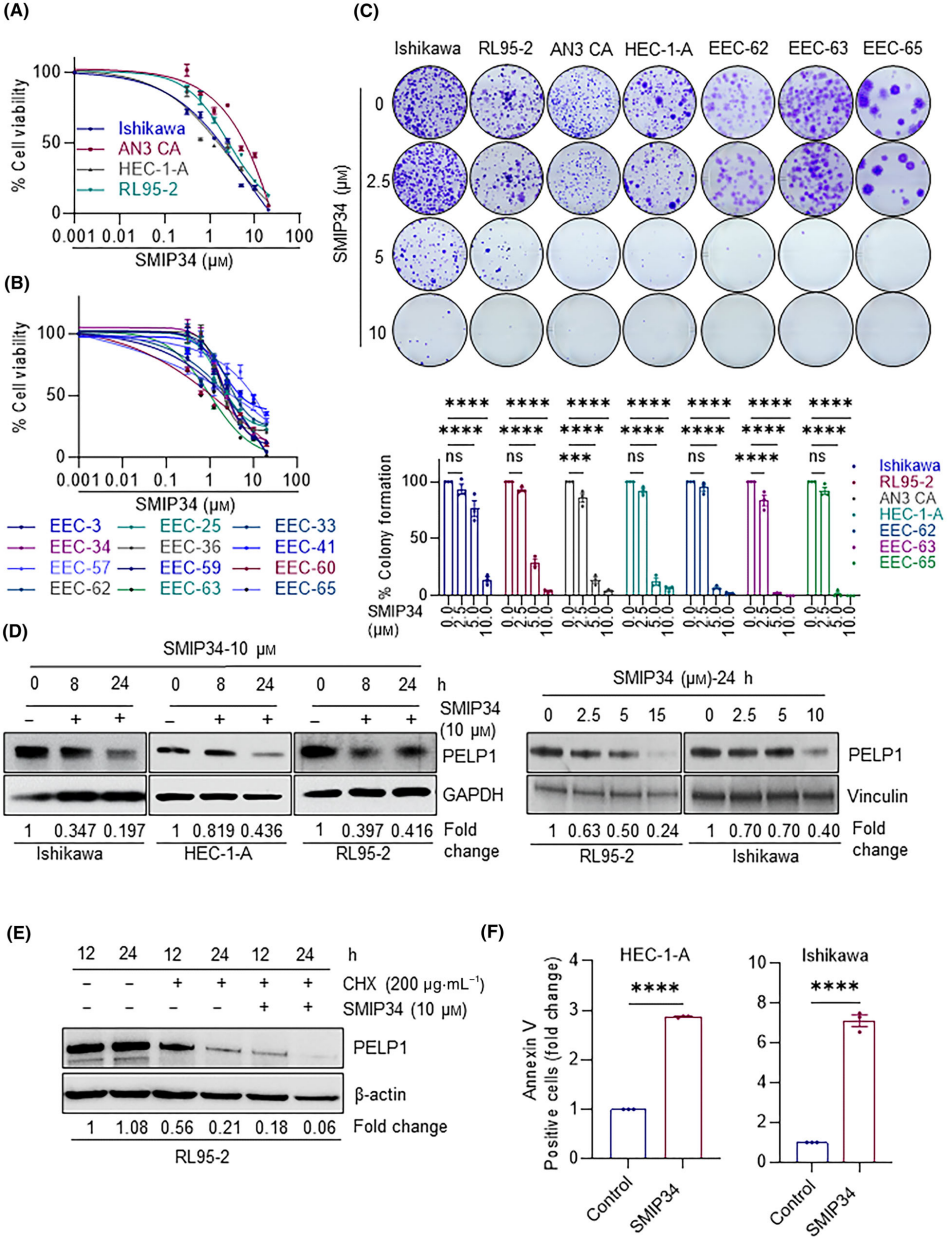
SMIP34 treatment decreased cell viability and increased apoptosis of ECa cells. After treating established (A) and primary (B) ECa cells with the indicated dose of SMIP34 for 7 days, the cell viability was assessed using the MTT assays. Results shown are from one of three experiments (*n* = 3), all of which had similar results. (C) Established and primary ECa cells were treated with the indicated doses of SMIP34, and the colony formation was examined after 14 days. The number of colonies were quantified, and the percentage of colonies were shown as bar graphs in the lower panel. Results shown are from one of three experiments (*n* = 3), all of which had similar results. Results show mean ± SEM, *n* = 3. *P* values are calculated using two‐way ANOVA, ***P < 0.001; ****P < 0.0001; ns, not significant. (D) ECa model cells were treated with SMIP34 (10 μm) for 8 and 24 h and levels of PELP1 was determined using Western blotting (Left panel). ECa model cells were treated with indicated concentrations of SMIP34 for 24 h and levels of PELP1 were determined using Western blotting (right panel). Results shown are from one of three experiments (*n* = 3), all of which had similar results. (E) PELP1 degradation was monitored using cycloheximide chase experiment. Western blotting was utilized to evaluate the expression of PELP1 in whole cell lysates. Results shown are from one of three experiments (*n* = 3), all of which had similar results. (F) ECa cells were treated with SMIP34 (20 μm), and the impact of this treatment on apoptosis was evaluated using an Annexin V/PI kit. Results shown are from one of three experiments (*n* = 3), all of which had similar results. Data are represented as mean ± SEM, *n* = 3. *P* values are calculated using *t*‐test, ****P < 0.0001.

A first‐in‐class small molecule PELP1 inhibitor, SMIP34, has been recently discovered by our group, and it binds to the PELP1 protein and promotes its degradation [27]. Using MTT cell viability assays, we investigated whether inhibiting PELP1 with SMIP34 decreases the viability of ECa cells. Results using 4 established and 12 primary ECa cells demonstrated that SMIP34 is highly effective in lowering ECa cell viability, with an IC_50_ of 1–5 μm (Fig. 2A,B). Similarly, SMIP34 is effective at reducing the colony formation ability of established and primary ECa cells (Fig. 2C). Western blotting analyses confirmed that SMIP34 treatment decreased PELP1 expression in a time‐ and dose‐dependent manner (Fig. 2D). We then used cycloheximide chase experiment to monitor the kinetics of PELP1 degradation. The results showed enhanced kinetics of degradation of PELP1 in the presence of cycloheximide, confirming SMIP34 promotes degradation of PELP1 (Fig. 2E). Additionally, SMIP34 treatment promoted apoptosis in ECa cells (Fig. 2F). Collectively, these findings imply that SMIP34 treatment effectively reduced cell viability and increased apoptosis of ECa cells.

### 3.3 PELP1 inhibition reduces the expression of genes involved in ribosome biogenesis and translation

**Fig. 3:**
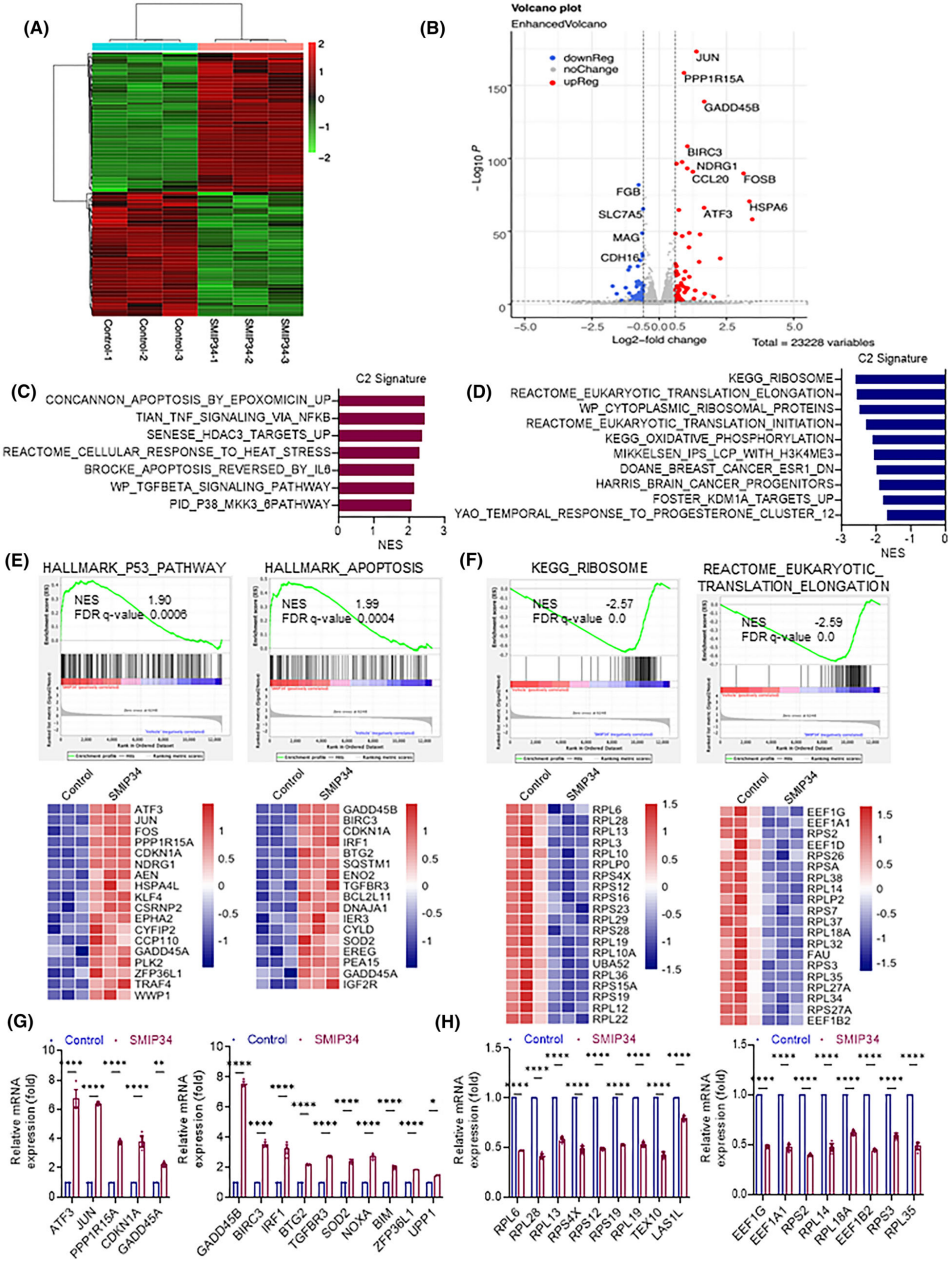
SMIP34 treatment elevated apoptosis pathways and downregulated ribosomal pathways. Total RNA was utilized for RNA‐seq after SMIP34 (20 μm) treatment on HEC‐1‐A cells for 24 h (*n* = 3). (A) Heatmap of the genes with differential expression is displayed. (B) Volcano plot of the genes with altered expression following SMIP34 treatment. (C, D) Upregulated and downregulated pathways were identified using differentially expressed genes using C2 signature. GSEA enrichment plots show SMIP34 regulated genes display positive enrichment with p53, and apoptosis pathways (E), and negative enrichment with the ribosome, and translation elongation pathways (F) with heatmap image of the specific genes in each pathway affected by SMIP34 treatment. The GSEA software was used to determine the *P* value, FDR *q* value, and normalized enrichment score (NES). (G, H) HEC‐1‐A cells were treated with either the vehicle (control) or SMIP34 for 24 h, and RT‐qPCR was used to validate the specific genes that were differentially regulated by SMIP34 treatment in RNA‐seq analysis. Data are represented as mean ± SEM. *P* values are calculated using two‐way ANOVA, *P < 0.05; **P < 0.01; ****P < 0.0001.

To understand the molecular mechanism(s) by which PELP1 inhibition reduces cell viability, we analyzed transcriptional alterations by RNA‐seq. Overall, 173 genes (with threshold value of Log_2_FC > 0.5 and with adjusted *P*‐value < 0.05) were differentially expressed in SMIP34‐treated cells. The heatmap (Fig. 3A) and volcano plot (Fig. 3B) display the differential expression profiles between control and SMIP34‐treated groups. The GSEA analysis showed a positive association between SMIP34‐regulated genes with the signatures of TNF, apoptosis, and TGF‐beta signaling, while a negative correlation was observed with the translation, and ribosomal genes (Fig. 3C,D). Importantly, we discovered that SMIP34‐regulated genes were positively enriched with gene sets of p53 and apoptosis pathway, and negatively enriched with gene sets for the ribosome and translation elongation (Fig. 3E,F). Further, validation tests using RT‐qPCR revealed that in ECa cells treated with SMIP34, genes involved in the p53 and the apoptosis pathways were elevated (Fig. 3G), whereas genes involved in ribosome biogenesis and translation elongation were downregulated (Fig. 3H). Collectively, these findings imply that pharmacological inhibition of PELP1 with SMIP34 significantly decreases expression of genes involved in protein translation, ribosomal biogenesis, and increases the expression of genes that induce apoptosis.

### 3.4 PELP1 inhibition reduces the levels of the proteins in the WDR18/PELP1 Rix1 complex

**Fig. 4:**
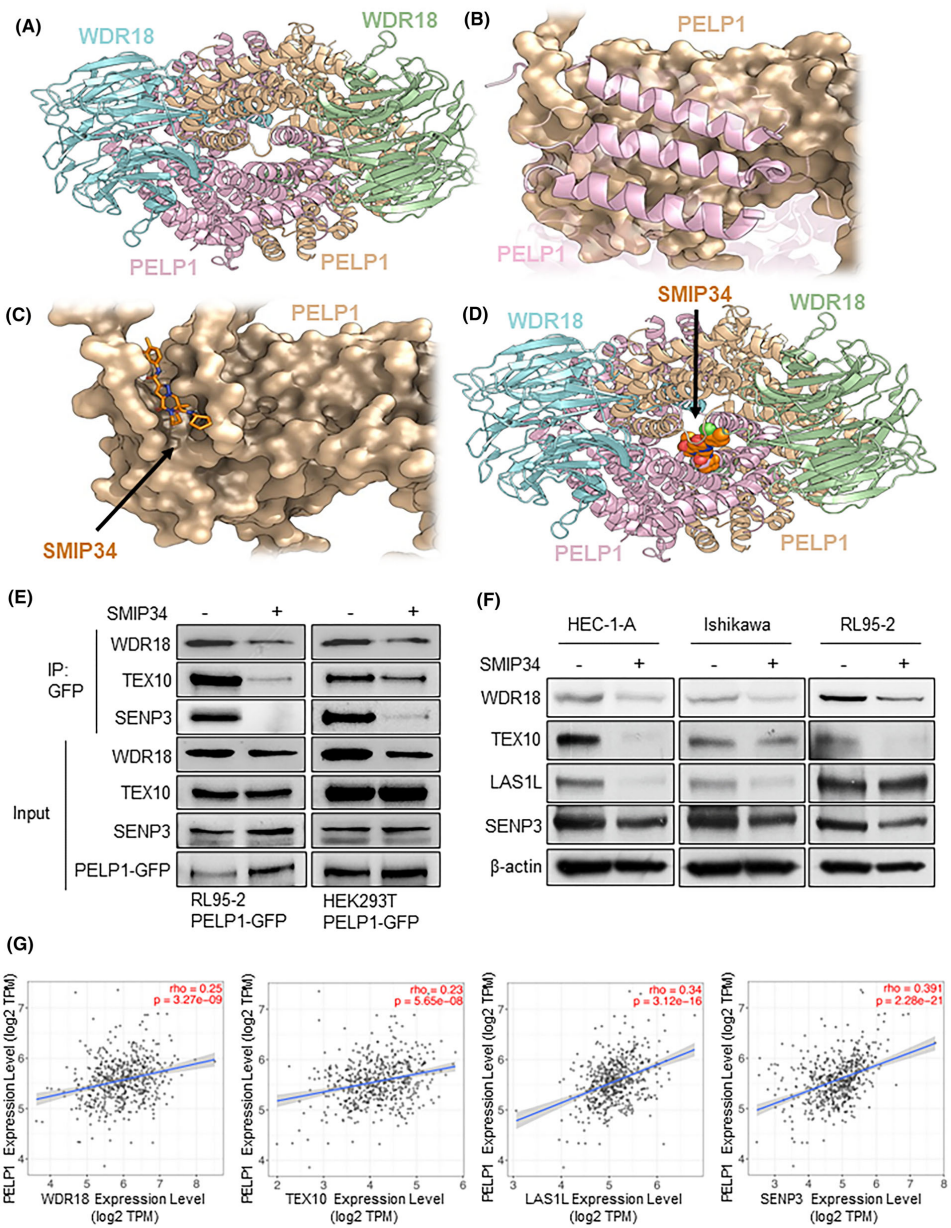
SMIP34 binding to PELP1 disrupts the formation of the Rix1 complex. (A) The CryoEM structure of WDR18/PELP1 Rix1 complex (PDB code 7UWF [33]) reveals the protein–protein interface between WDR18 and the PELP1 homodimer, which suggests that homodimerization of PELP1 is essential for WDR18 binding. (B) Local view of the homodimerization interface of PELP1 in WDR18/PELP1 Rix1 complex. Several key helices are involved in the homodimerization of PELP1 (*n* = 1). (C) According to the full‐length PELP1 monomer structure predicted by AlphaFold2, the loop region of #696–720 binds to the homodimerization interface and constitutes the SMIP34 binding site [27], which suggests that SMIP34 selectively binds to PELP1 monomer against dimer. (D) Superimposition of SMIP34 to the WDR18/PELP1 Rix1 complex suggests that SMIP34 binding is expected to competitively inhibit PELP1 homodimerization and Rix1 complex formation. (E) RL95‐2‐PELP1‐GFP, and HEK293T‐PELP1‐GFP cells were treated with vehicle (control) or SMIP34 (15 μm, 6 h), PELP1‐GFP was immunoprecipitated using GFP Trap beads and the ability of PELP1‐GFP to interact with Rix1 complex proteins was visualized using Western blotting. Results shown are from one of three experiments (*n* = 3), all of which had similar results. (F) Western blotting was used to assess the status of the Rix1 complex proteins after treatment with SMIP34 (10 μm, 24 h) in ECa model cells. Results shown are from one of three experiments (*n* = 3), all of which had similar results. (G) Gene correlation analysis of PELP1 with various components of Rix1 complex proteins in ECa using TCGA database.

Based on the newly released Cryo‐EM structure of the WDR18/PELP1 Rix1 complex (PDB code 7UWF) [33], the PELP1 homodimer acts as the core of the WDR18/PELP1 assembly. As depicted in Fig. 4A, each WDR18 subunit in the complex directly interacts with both PELP1 subunits, which suggests that PELP1 dimerization is essential for forming WDR18/PELP1 complex. PELP1 forms the symmetric homodimer mainly with α helices 17, 20, and 22 of the Rix1 domain (Fig. 4B). Based on our previous data [27], SMIP34 could bind to the same region involved in the PELP1 homodimerization (Fig. 4C,D), which is likely to disrupt the formation of PELP1 homodimer and subsequently block the formation of the WDR18/PELP1 Rix1 complex. In particular, according to the AlphaFold [34] predicted structure of full‐length PELP1 monomer, the loop region containing amino acids #696–720 may bind to the homodimer interface and constitutes the SMIP34 binding site. This information suggests that SMIP34 could selectively bind to PELP1 monomer against dimer. Moreover, reduced dimerization of PELP1 may decrease the stability of PELP1, which could be a potential reason for SMIP34‐induced PELP1 degradation observed in our study. In addition to alteration in the conformation of #696–720 loop, the binding of SMIP34 may influence the conformation of the following C‐terminal region, which may disturb the interaction of other PELP1 partners such as SENP3 and TEX10 binding to the C‐terminal region. We performed IP assays utilizing PELP1‐GFP‐stably expressed RL95‐2 and HEK293T cells to test this theory. To prevent PELP1 from degrading, we employed SMIP34 treatment for a brief (6 h) period, and GFP‐TRAP beads were used for the IP. The results from IP experiments demonstrated decreased association of Rix1 complex proteins WDR18, TEX10, and SENP3 with PELP1 under conditions of SMIP34 treatment (Fig. 4E). Our earlier studies using PELP1 knockout models and mass spectrometry analyses suggested that PELP1 is essential for the stability of the Rix1 complex [35]. We therefore used Western blotting analysis to determine the status of the Rix1 complex proteins under conditions of SMIP34 treatment. Western blotting analysis demonstrated that SMIP34 treatment reduces the levels of WDR18/PELP1 Rix1 complex proteins WDR18, TEX10, LAS1L, and SENP3 in ECa cell lines (Fig. 4F). Analysis of TCGA ECa patients' datasets showed that PELP1 expression is positively correlated with WDR18, TEX10, LAS1L, and SENP3 (Fig. 4G). These results suggest that PELP1 inhibitor SMIP34 disrupts the assembly of WDR18/PELP1 Rix1 complex.

### 3.5 PELP1 inhibition by SMIP34 reduced levels of rRNA and new protein synthesis

**Fig. 5:**
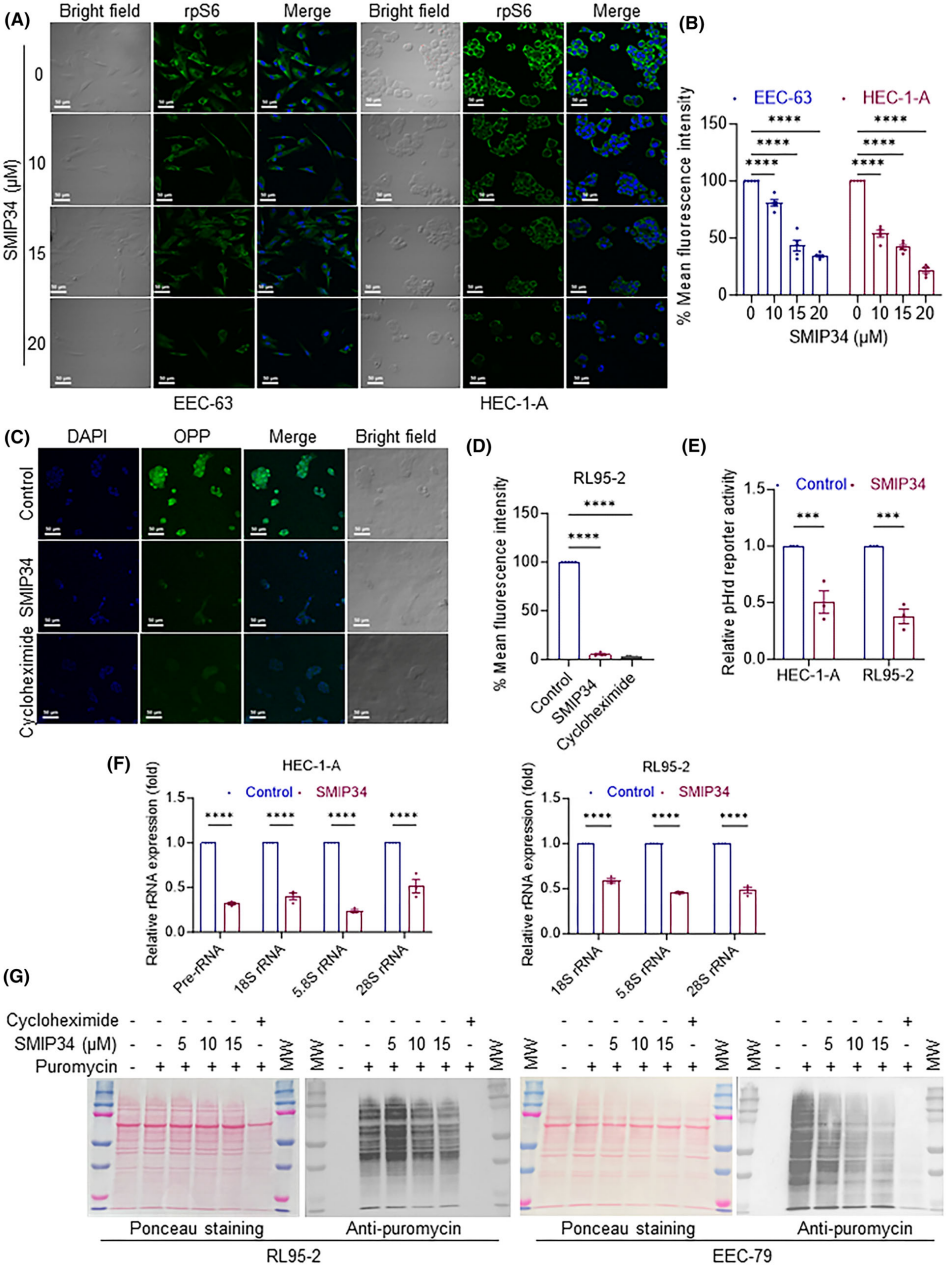
SMIP34 treatment reduced ribosomal biogenesis and new protein synthesis. (A) EEC‐63, and HEC‐1‐A cells were treated with vehicle (control) or SMIP34 (10, 15, 20 μm) for 16 h and status of 40S ribosomal subunit protein rpS6 was visualized using confocal microscopy. Results shown are from one of two experiments (*n* = 2), and both had similar results (scale bar 50 μm). (B) The quantification of total fluorescence intensity was analyzed using imagej and the percentages of mean fluorescence intensity (total fluorescence intensity divided by the cell counting in each field) normalized to control group were shown as bar graphs. Data are represented as mean ± SEM. *P* values are calculated using two‐way ANOVA, ****P < 0.0001. (C) RL95‐2 cells were treated with the SMIP34 (15 μm, 16 h), followed by incubation with OPP (90 min), a puromycin analog that incorporated only to newly synthesized proteins and the effect of the treatment on new protein synthesis was monitored by reduced FITC signal using confocal microscopy (scale bar 50 μm). Results shown are from one of two experiments, and both had similar results. (D) The quantification of total fluorescence intensity was analyzed using imagej and the percentages of mean fluorescence intensity (total fluorescence intensity divided by the cell counting in each field) normalized to control group were shown as bar graphs. Data are represented as mean ± SEM. *P* values are calculated using two‐way ANOVA, ****P < 0.0001. (E) HEC‐1‐A or RL95‐2 cells were transfected with pHrD Luciferase reporter vector. After 24 h, cells were treated with vehicle or SMIP34 (15 μm, 16 h) and the pHrD reporter gene activity was measured. Results shown are from one of four experiments, all of which had similar results. Data are represented as mean ± SEM. *P* values are calculated using two‐way ANOVA, ***P < 0.001. (F) HEC‐1‐A or RL95‐2 cells were treated with vehicle or SMIP34 (15 μm, 16 h) and the levels of pre‐RNA, 18S, 5.8S, 28S rRNA was measured using RT‐qPCR using Actin as internal control. The results shown are from one of three experiments (*n* = 3), all of which had similar results. Data are represented as mean ± SEM. *P* values are calculated using two‐way ANOVA, ****P < 0.0001. (G) RL95‐2 and EEC‐79 cells were treated with the SMIP34 (5, 10, 15 μm), followed by 30 min incubation with puromycin (1 μm) and the effect of the treatment on global protein synthesis was measured by Western blotting using anti‐puromycin antibody. MW represents molecular weight maker. The results shown are from one of five experiments, all of which had similar results.

To determine whether blocking of PELP1 functions in Rix complex with SMIP34 treatment decreases ribosomal biogenesis, we investigated the expression of the 40S ribosomal subunit protein rpS6 as a ribosomal marker. After 16 h of exposure to various concentrations of SMIP34, EEC‐63 and HEC‐1‐A cells were fixed, and stained with rpS6 antibody. In comparison to vehicle‐treated control, SMIP34 treatment decreased rpS6 levels in a dose‐dependent manner (Fig. 5A,B). We then used the cell‐permeable, alkyne‐containing puromycin analog OPP to investigate whether decreased ribosomal biogenesis is correlated to decreased new protein synthesis. In this assay, the truncated C‐terminal alkyne‐labeled proteins were detected via copper‐catalyzed click chemistry using 5 FAM‐Azide. After 16 h of SMIP34 treatment, RL95‐2 cells were treated with OPP for 90 min. The cells were then prepared for florescence labeling to detect protein production and visualized by confocal microscopy. Results showed that SMIP34 treatment substantially reduced 5‐FAM fluorescence, indicating decreased protein synthesis (Fig. 5C,D). Pre‐rRNA processing and synthesis are crucial and rate‐limiting phases in the ribosomal biogenesis process. Since earlier studies indicated that PELP1 regulates rRNA transcription [36], we examined whether SMIP34 treatment regulate rRNA transcription. To test this, we have used the human rDNA‐promoter luciferase reporter (pHrD‐IRES‐Luc) assay [36]. The results using both HEC‐1‐A and RL95‐2 cells showed that SMIP34 treatment significantly decreased the activity of the rDNA‐promoter luciferase reporter (Fig. 5E). We then examined the status of rRNAs upon SMIP34 treatment using RT‐qPCR. Our findings demonstrated that treatment with SMIP34 significantly decreased the quantities of mature 18S, 5.8S, and 28S rRNAs as well as pre‐rRNA (Fig. 5F). The relationship between new protein synthesis and SMIP34 treatment was then investigated by subjecting RL95‐2 and EEC‐79 cells to varying concentrations of SMIP34, puromycin‐incubation, and performing Western blotting analyses of total lysates with anti‐puromycin antibody. As expected, the administration of SMIP34 significantly decreased global protein production in a dose‐dependent manner (Fig. 5G). Cycloheximide treatment was used as positive control and it also blocked new protein synthesis in these assays. Collectively, these findings show that SMIP34 impairs levels of rRNA, which in turn in decreases new protein synthesis.

### 3.6 PELP1 inhibition enhanced the therapeutic efficacy of mTOR inhibitors

**Fig. 6:**
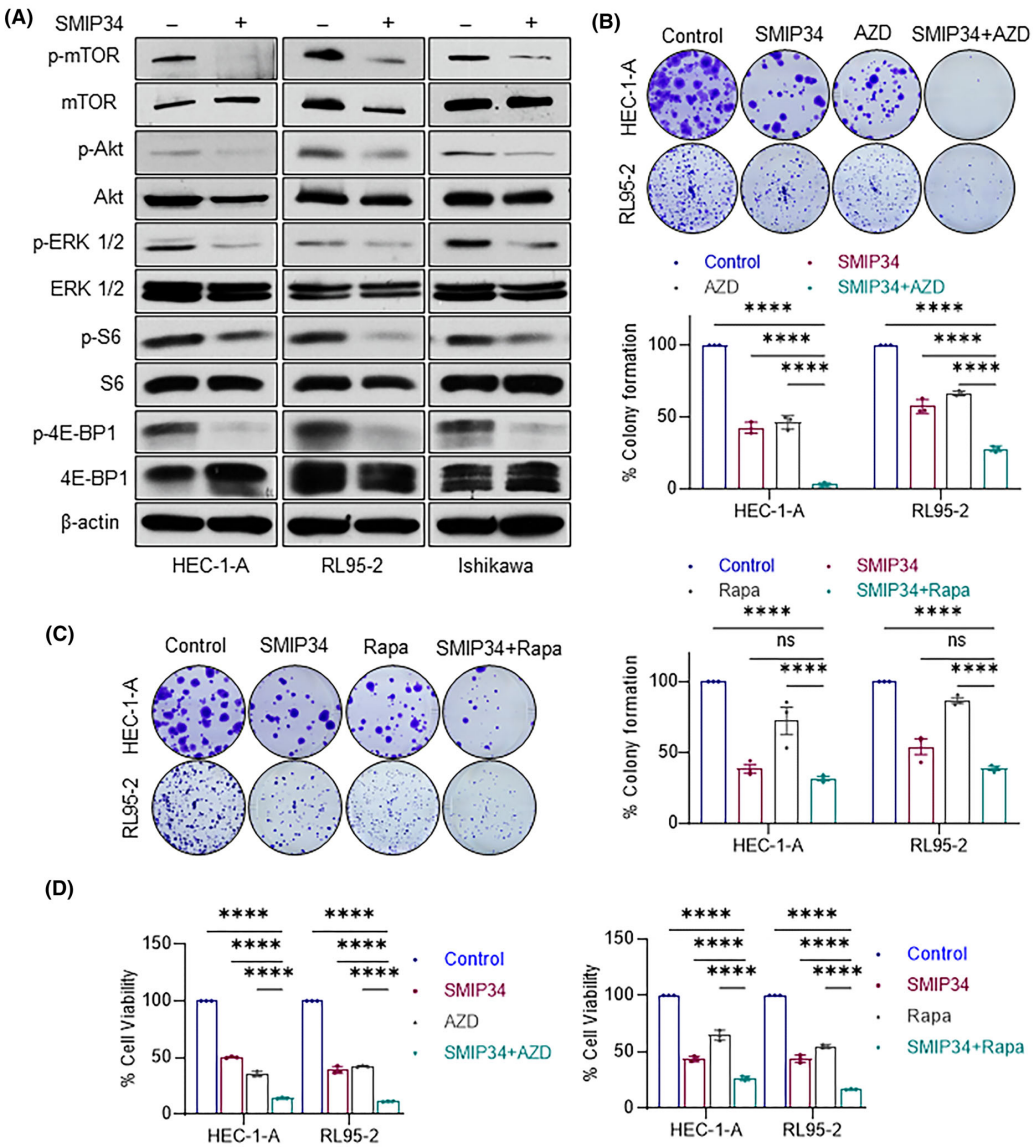
SMIP34 treatment blocked PELP1‐mediated extranuclear signaling and enhanced the therapeutic efficacy of mTOR inhibitors. (A) HEC‐1‐A, RL95‐2, and Ishikawa cells were treated with vehicle (control) or SMIP34 (10 μm) and status of known PELP1 downstream signaling was analyzed using Western blotting. The results shown are from one of three experiments (*n* = 3), all of which had similar results. (B, C) HEC‐1‐A, and RL95‐2 cells were treated with the SMIP34, mTOR inhibitors (Rapamycin and AZD8055) alone or in combination and the effect of the treatment on colony formation was examined after 14 days. The number of colonies were quantified, and the percentage of colonies were shown as bar graphs. The results shown are from one of two experiments (*n* = 2), and both had similar results. Results show mean ± SEM. *P* values are calculated using two‐way ANOVA, ****P < 0.0001; ns, not significant. (D) HEC‐1‐A, and RL95‐2 cells were treated with SMIP34, mTOR inhibitors alone or in combination and the effect of the treatment on cell viability was measured using MTT assays. The results shown are from one of two experiments (*n* = 2), both had similar results. Results show mean ± SEM. *P* values are calculated using two‐way ANOVA, ****P < 0.0001.

Endometrioid endometrial cancers are known to harbor mutations in PI3K/AKT/mTOR pathway genes. Further, previous research revealed that PELP1 oncogenic functions also entail direct contacts to modulate mTOR, pSrc‐MAPK, and PI3K‐AKT activities. Therefore, we investigated whether the state of these signaling pathways was changed due to the suppression of PELP1 signaling by SMIP34. Western blotting results showed that SMIP34 treatment dramatically decreased the activation status of AKT, ERK, mTOR and its downstream effectors, such as S6 and 4EBP1. Since we found a considerable downregulation of the mTOR signaling in cells treated with SMIP34 (Fig. 6A) and because EEC frequently exhibits changes in the PI3K/AKT/mTOR pathway [9, 37, 38], we examined whether SMIP34 treatment enhances the efficacy mTOR inhibitors. In comparison to a single agent, the combination of SMIP34 and mTOR inhibitors (AZD8055 or Rapamycin) is more potent in reducing the clonogenic survival (Fig. 6B,C) and cell viability (Fig. 6D) of HEC‐1‐A and RL95‐2 cells compared to monotherapy.

### 3.7 PELP1 inhibitor SMIP34 reduced the proliferation of patient‐derived ECa organoids and explants *ex vivo*

**Fig. 7:**
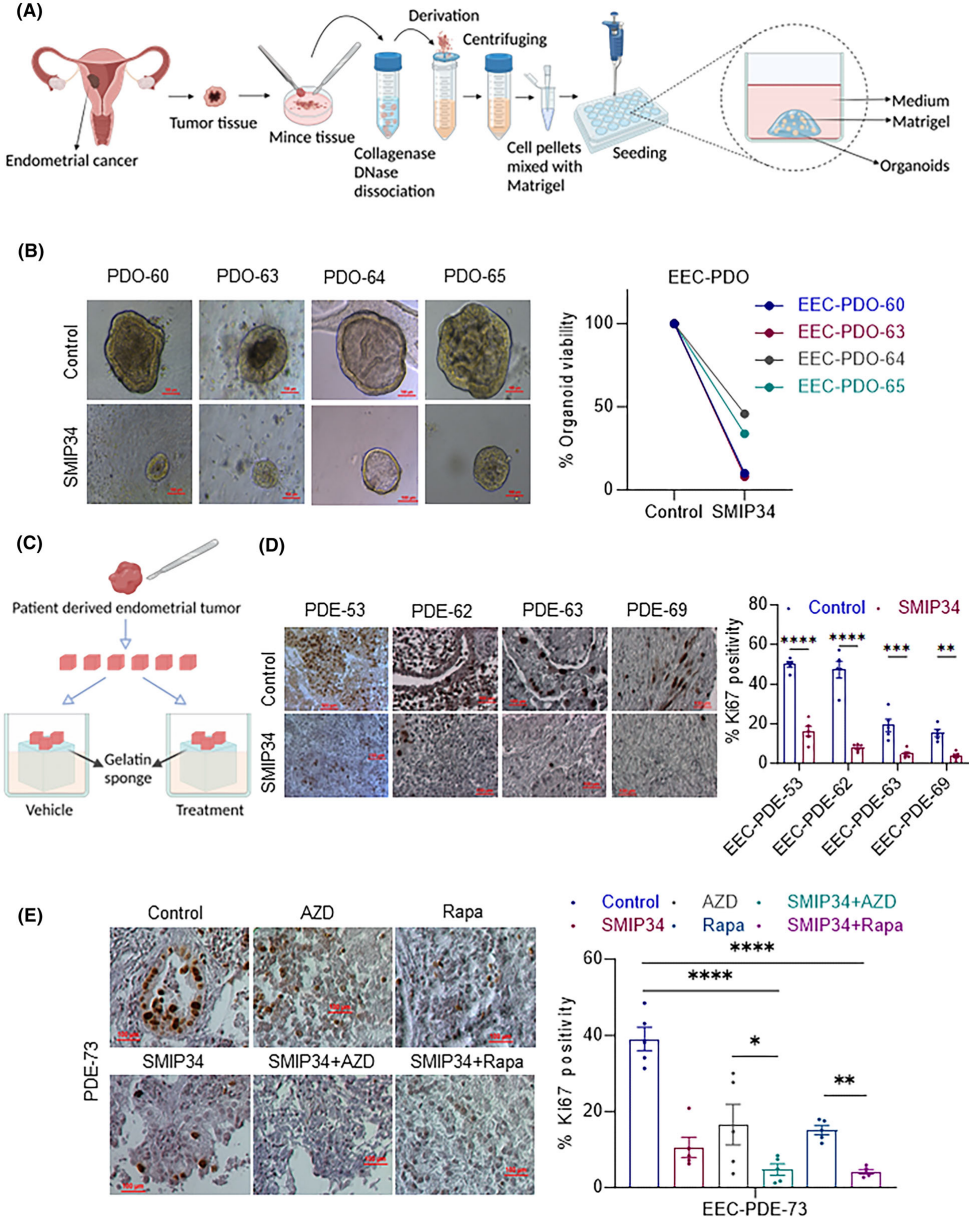
PELP1 inhibition using SMIP34 effectively inhibits the growth of PDOs and PDEs *ex vivo*. (A) Schematic figure of the procedure used for establishing organoids. (B) Using 3D CellTiter‐Glo assays, the impact of SMIP34 treatment on the viability of PDOs was evaluated. Representative pictures of PDOs cultured with or without SMIP34 treatment were shown (scale bar 100 μm). The results shown are from one of two experiments (*n* = 2), and both had similar results. (C) Schematic representation of PDE culture model. (D) ECa tumor tissues were cultured as explants and were treated with SMIP34 for 72 h. SMIP34's impact on the proliferation was analyzed by IHC studies using proliferation marker Ki67. Representative images from each explant tumor were shown on the left panel and the quantitation of Ki67 staining was shown on the right panel (scale bar 100 μm). The results shown are from one of four experiments (*n* = 4), all of which had similar results. Results show mean ± SEM. *P* values are calculated using two‐way ANOVA, **P < 0.01; ***P < 0.001; ****P < 0.0001. (E) Using IHC analysis, the effect of combination therapy of SMIP34 with mTOR inhibitors (AZD8055 or rapamycin) on PDE was evaluated using proliferation marker Ki67. Representative images from each explant tumor were shown on the left panel and the quantitation of Ki67 staining was shown on the right panel (scale bar 100 μm). The results shown are from one of three experiments (*n* = 3), all of which had similar results. Results show mean ± SEM. *P* values are calculated using two‐way ANOVA, *P < 0.05; **P < 0.01; ****P < 0.0001.

Using primary human ECa organoids, which were generated using previously described techniques [14], we next evaluated the effect of PELP1 inhibitor SMIP34 on ECa (Fig. 7A). Organoid viability assays revealed that in comparison to vehicle treatment (control), SMIP34 significantly decreased the viability of organoids (Fig. 7B). The effect of SMIP34 was further evaluated using an explant assay utilizing primary ECa tumor tissues. In this assay, surgically removed ECa tumors were cultured for a brief period in the presence of either SMIP34 or a vehicle (control) (Fig. 7C). Compared to tumors treated with a vehicle (control), ECa explants treated with SMIP34 showed a significant reduction in cell proliferation (Ki67 positive) (Fig. 7D). We then examined the impact of combining SMIP34 with mTOR pathway inhibitors. In ECa explants, the combination of SMIP34 with AZD8055 or Rapamycin is more effective than a single drug in suppressing tumor cell proliferation as indicated by Ki67 (Fig. 7E).

### 3.8 PELP1 inhibition by SMIP34 reduced ECa xenograft tumor growth *in vivo*

**Fig. 8:**
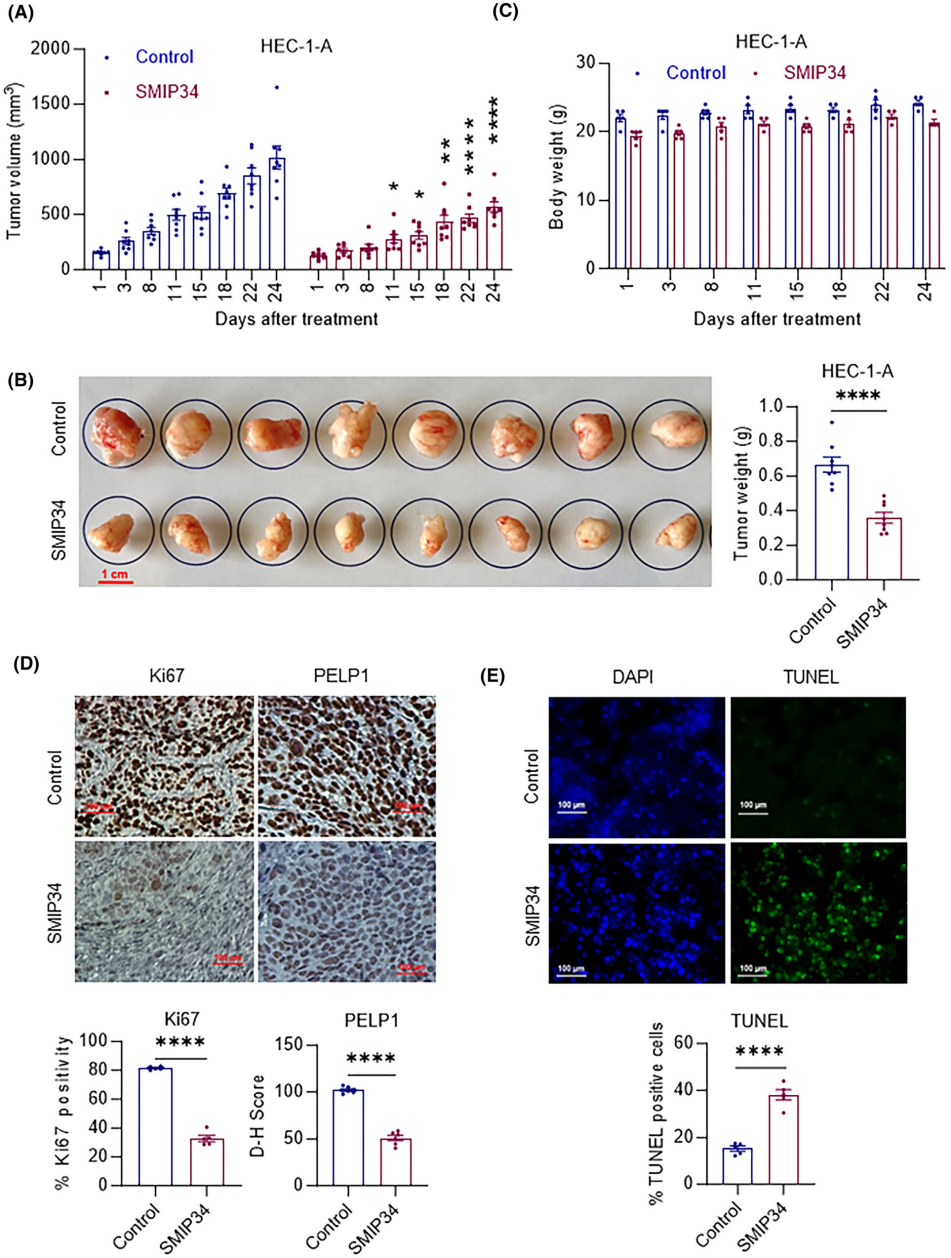
SMIP34 treatment suppresses EEC xenograft tumor growth *in vivo*. (A) HEC‐1‐A cells were injected subcutaneously into SCID mice. Following tumor establishment, mice were randomly assigned to receive either vehicle (control) or SMIP34 (20 mg·kg^−1^ body weight) 5 days a week through i.p. injection (*n* = 8). Tumor volume was assessed at 3–5 days intervals. *P* values are calculated using two‐way ANOVA, *P < 0.05; **P < 0.01; ****P < 0.0001. (B) Pictures of vehicle (control) and SMIP34‐treated tumors were displayed on the left panel and the weights of tumors were shown on the right panel (scale bar 1 cm). (C) The body weights of vehicle (control) and SMIP34‐treated mice were shown. Tumor samples from the vehicle (control) and SMIP34 treatment groups were processed by IHC for Ki67 (proliferation marker), PELP1 staining (D), and TUNEL assay (apoptosis marker) (E) and quantitated (scale bar 100 μm). The results shown are from one of two experiments (*n* = 2), and both had similar results. Representative images were shown on the upper panel and quantitation of the staining were shown on the lower panel. Data represented as mean ± SEM. *P* values are calculated using *t*‐test, ****P < 0.0001.

To assess the effectiveness of SMIP34 *in vivo* on ECa tumor growth, we created HEC‐1‐A xenograft tumors in the flanks of SCID mice. Mice with HEC‐1‐A xenografts were randomly assigned to receive daily i.p. doses of the vehicle (0.3% hydroxypropyl cellulose) or SMIP34 (20 mg·kg^−1^), 5 days per week according to our previously published studies [27]. When compared to control, SMIP34 treatment significantly reduced the tumor growth (Fig. 8A) and reduced the tumor's weight (Fig. 8B). The body weights of the mice in the SMIP34 treatment group and the control group did not change significantly (Fig. 8C). Additionally, compared to vehicle‐treated ECa tumors (control), SMIP34 treated ECa tumors had decreased cell proliferation (Ki67 staining), PELP1 expression (Fig. 8D) and increased apoptosis (TUNEL fluorescence) (Fig. 8E). These findings collectively imply that SMIP34 decreases the growth of ECa xenograft tumors *in vivo*.

## 4 Discussion

Endometrial carcinoma most frequently affects older women and the prevalence of ECa is anticipated to rise due to increase in the elderly population. The overall mortality rate from ECa has not decreased recently, however, the outcome for recurrent ECa is unfavorable. This is notably relevant to the 50% of women who report with an additional recurrence of pelvic disease. There is an urgent need for new therapeutic options for ECa, specifically new therapeutic targets and agents are needed to support current ECa‐directed therapy. The results from this study suggest that PELP1 expression is upregulated in ECa. Using knockdown and pharmacological inhibition, we demonstrated that PELP1 may be a novel target for ECa and inhibition of PELP1 decreases PI3K‐AKT‐mTOR signaling, and ribosomal biogenesis.

PELP1 oncogenic signaling has been linked to the progression of many cancers [20]. According to published studies, PELP1 may act as a biomarker for tumors with poor prognoses, such as breast and prostate cancer [39]. In a prior work, PELP1 expression in ECa cells and aberrant PELP1 expression in ECa tumors was reported [16]. Another study discovered that PELP1 knockdown reduces the ability of ECa cells to migrate and invade [40]. Our IHC study employing a TMA also showed that PELP1 was expressed more strongly in ECa tumor tissue than in normal endometrium. Additionally, the findings from the TNMplot database supported the upregulation of PELP1 in the ECa. Collectively, these findings indicated that PELP1 dysregulation might occur in ECa; however, additional research involving more patient tumors is required to confirm this finding and establish PELP1 as a predictive biomarker and therapeutic target for ECa.

Recent research has shown that the first‐in‐class PELP1 inhibitor SMIP34 is effective at delaying the progression of breast cancer [27]. However, the role of PELP1 and the utility of SMIP34 blocking ECa progression remain unknown. In this investigation, we used several established and primary ECa cells to discover that SMIP34 reduced PELP1 expression, decreased cell viability and colony formation, and increased apoptosis. RNA‐seq analyses demonstrated that pharmacological PELP1 inhibition leads to suppression of the ribosomal and translation pathways and activation of the p53 and apoptosis pathways. Further, using patient‐derived ECa organoids and explants, and xenograft models, our studies showed that SMIP34 is effective both *ex vivo* and *in vivo* to suppress the tumor growth. These findings concur with the DepMap data, which shows that PELP1 knockout decreases the survival of ECa cells.

The PELP1‐TEX10‐WDR18 complex functions as a ribosomal biogenesis regulator and PELP1 localization is shown to regulate the rate of ribosomal synthesis [41]. PELP1 localizes to the nucleolus and plays a role in optimal production of the 28S rRNA [42]. Through the controlled recruitment and release of the AAA ATPase MDN1, PELP1 has been demonstrated to act as a regulatory point for mammalian 60S maturation [43]. Recent study using CRISPR PELP1 knockout model cells and global analysis showed that PELP1‐mediated oncogenic functions involve control of several transcription factors including c‐Myc and ribosomal biogenesis. In our RNA‐seq experiments, we discovered that pharmacological PELP1 inhibition suppresses the expression of several ribosomal genes. Our RNA‐seq data further indicated that PELP1 is also critical for the elongation of protein translation. Our mechanistic studies and puromycin labelling studies also confirmed that SMIP34 treatment contributes to decreased ribosomal biogenesis and protein synthesis. Reporter assays confirmed that SMIP34 treatment decreased rDNA‐promoter luciferase reporter activity. Similarly, RT‐qPCR analyses confirmed that SMIP34 treatment significantly decreased the quantities of mature 18S, 5.8S, and 28S rRNAs as well as pre‐rRNA. Collectively, these findings suggest that SMIP34‐mediated growth inhibitory effects are in part mediated by blocking PELP1 facilitated functions in rRNA synthesis and protein synthesis. Earlier studies showed that interaction between PELP1 and mTOR led to the stimulation of mTOR downstream signaling when PELP1 was dysregulated [44]. Interestingly, downregulation of a subset of genes related to ribosomal and translation pathway (TOP mRNAs) could be resulted from perturbed mTOR signaling by SMIP34‐mediated downregulation of PELP1 expression and this may also impact reduced ribosomal biogenesis in SMIP34 treated cells. Future mechanistic studies are clearly needed to differentiate these two effects and such studies are beyond the scope of this investigation.

The structure of the human PELP1‐Rix1 domain coupled to WDR18 was established in recent research utilizing cryo‐electron microscopy (cryo‐EM) at a resolution of 2.7 Å [33]. Based on the newly released Cryo‐EM structure of the WDR18/PELP1‐Rix1 complex (PDB code 7UWF) [33], the PELP1 homodimer acts as the core of the WDR18/PELP1 assembly. Based on our previous study [27], SMIP34 could bind to the same region involved in the PELP1 homodimerization, which is likely to disrupt the formation of PELP1 homodimer and subsequently block the formation of WDR18/PELP1‐Rix1 complex. Moreover, reduced dimerization of PELP1 may decrease the stability of PELP1, which could be a potential reason for the SMIP34 induced PELP1 degradation observed in our study. In the present study, we discovered that SMIP34 is efficient at preventing the PELP1‐Rix1 complex. Additionally, the Rix1 complex proteins such as WDR18, TEX10, LASIL, and SENP3 had their levels markedly decreased after treatment with SMIP34. These findings are consistent with previously published studies and support the theory that the connection of PELP1 with the Rix1 complex contributes to the stability of this complex.

Although PELP1 does not have any known enzymatic activity, it may serve as a protein scaffold that aids in the assembly of signaling complexes. For instance, PELP1 has several proline‐rich regions that contain consensus PXXP motifs. These motifs enable PELP1 to interact with Src and the P85 regulatory subunit of PI3K [45]. The c‐Src‐PI3K‐ERK pathway had lower expression in gastric cancer models when PELP1 was suppressed [45]. Further, in ovarian cancer cells, c‐Src and AKT signaling was diminished when PELP1 was downregulated [15]. Breast cancers with migratory characteristics are caused by PELP1 being localized in the cytoplasm, which increases pro‐tumorigenic IKK and NF‐κB signaling [46]. The interaction between PELP1 and mTOR led to the stimulation of mTOR downstream signaling when PELP1 was dysregulated [44]. In line with previous research, we discovered that treatment with SMIP34 dramatically decreased the activity of the AKT, ERK, and mTOR in ECa cells. It is known that the PI3K/AKT/mTOR pathway is altered at the molecular level in EEC [9, 37, 38, 47]. According to our findings, mTOR inhibitors were more effective at lowering the viability of ECa cells when PELP1 was also inhibited by SMIP34. A combination therapy of SMIP34 and mTOR inhibitors may boost their efficacy in treating ECa because ongoing clinical trials have demonstrated mTOR inhibitors' moderate effectiveness in treating ECa.

## 5 Conclusions

In conclusion, our findings showed that PELP1 suppression decreased ECa cell viability both *in vitro, ex vivo* and *in vivo*. Mechanistic investigations revealed that the stability of the Rix1 complex formation is impacted by PELP1 inhibition, which lowers ribosomal biogenesis. PELP1 could serve as a potential therapeutic target and that SMIP34 may be a novel class of drug for treating ECa.

## Conflict of interest

The authors declare no conflict of interest.

## Author contributions

RKV, SV, and GRS designed the experiments and interpreted the results. XY, ZeL, WT, UPP, ABC, KAA, RG, and XL, conducted the experiments; YY and DZ designed and interpreted the modeling studies. RKV, XY, ZhL, and YC designed and intercepted the results of RNA‐seq. ERK and PTV designed and intercepted the results of TMA, PDO, and PDE studies. SV and XY designed and conducted xenograft studies; RKV, XY, and SV wrote the manuscript.

### Peer review

The peer review history for this article is available at https://www.webofscience.com/api/gateway/wos/peer‐review/10.1002/1878‐0261.13539.

## Supporting information


**Table S1.** List of the primers used in the study.

**Table S2.** Endometrial tumor tissue Demographic Information.

## Data Availability

All data generated for this study is included in this article. The GEO database contains RNA‐seq data that has been deposited with a GEO accession number (GSE226850).
