# Supplementary material for: PELP1 inhibition by SMIP34 reduces endometrial cancer progression via attenuation of ribosomal biogenesis
Source: Mol Oncol. 2023 Nov 1;18(9):2136–56. doi: 10.1002/1878-0261.13539 (PMC11467795; doi:10.1002/1878-0261.13539)
Supplement: Supplementary file 1 — Table S1. List of the primers used in the study. [file MOL2-18-2136-s002.pdf]

List of the primers used in the study

Gene Name	Forward	Reverse
ATF3	CCTCTGCGCTGGAATCAGTC	TTCTTTCTCGTCGCCTCTTTTT
JUN	TCCAAGTGCCGAAAAAGGAAG	CGAGTTCTGAGCTTTCAAGGT
PPP1R15A	ATGATGGCATGTATGGTGAGC	AACCTTGCAGTGTCCTTATCAG
CDKN1A	TGTCCGTCAGAACCCATGC	AAAGTCGAAGTTCCATCGCTC
GADD45A	CTCAACGTCGACCCCGATAA	GCCTGGATCAGGGTGAAGTG
GADD45B	TACGAGTCGGCCAAGTTGATG	GGATGAGCGTGAAGTGGATTT
BIRC3	CCAAGTGGTTTCCAAGGTGT	TCTCCTGGGCTGTCTGATGT
IRF1	CTGTGCGAGTGTACCGGATG	ATCCCCACATGACTTCCTCTT
BTG2	ACCACTGGTTTCCCGAAAAG	CTGGCTGAGTCCGATCTGG
TGFBR3	GTGTTCCCTCCAAAGTGCAAC	AGCTCGATGATGTGTACTTCCT
SOD2	GCTCCGGTTTTGGGGTATCTG	GCGTTGATGTGAGGTTCCAG
NOXA	GCTGGAAGTCGAGTGTGCTA	CCTGAGCAGAAGAGTTTGGA
BIM	TGGCAAAGCAACCTTCTGATG	GCAGGCTGCAATTGTCTACCT
ZFP36L1	TCCAGCATAGCTTTAGCTTTGC	GGTCATCGGCGCTCAGAATAG
UPP1	TGATTGCCCCGTCAGACTTTT	CACCAACGCACCTGATGAAG
RPL6	ATTCCCGATCTGCCATGTATTC	TACCGCCGTTCTTGTCACC
RPL28	GCATCTGCAATGGATGGTCG	GTCCGTTGTAGCGGAAGGAA
RPL13	TCAAAGCCTTCGCTAGTCTCC	GGCTCTTTTTGCCCGTATGC
RPS4X	GAGGAGGCCAAGTACAAGTTG	CACAGGTTACCAGTGTCGAAC
RPS12	TGCTGGAGGTGTAATGGACG	GGCGCTTGTCTAAGGCTTTG
RPS19	AAGCTGAAAGTCCCCGAATGG	AGTTCTCATCGTAGGGAGCAAG
RPL19	GGGCATAGGTAAGCGGAAGG	TCAGGTACAGGCTGTGATACA
TEX10	GAAGTGGAGACTTGTGACAAAGG	AGGTGAAGAGTGCTGGACAAT
LAS1L	GTGTGGAGTGCGTGGTACG	TGCAACTTATGGTCGTCACAG
EEF1G	AACCGCACCCCTGAATTTCTC	GGCGTTGCTCTCAAACACAC
EEF1A1	TGTCGTCATTGGACACGTAGA	ACGCTCAGCTTTCAGTTTATCC
RPS2	GGCCTCTCTCAAGGATGAGGT	GTCCCCGATAGCAACAAATGC
RPL14	GACCTTGCACTCAAGTGAGGA	CTTGTCGGACATACTTCTGGTG
RPL18A	ATCTTTGCGCCTAATCATGTCG	CCACAGTAGACAATCTCCCCT
EEF1B2	GGTGCTCAACGATTACCTGG	ATACCAACGTAGGGCATGACA
RPS3	AGGGCAGTGTAGAGCTTTATGC	ATGAACCGCAGCACACCATAG
RPL35	AGCTCTCTAAGATCCGAGTCG	GAACACGGGCAATGGATTTCC
Human pre-rRNA	TGTCAGGCGTTCTCGTCTC	AGCACGACGTCACCACATC
18S rRNA	CTACTTGGATAACTGTGGTA	CGAGGTTATCTAGAGTCAC
5.8S rRNA	AGCGCTAGCTGCGAGAATTA	GACGCTCAGACAGGCGTAG
28S rRNA	CCGCTGCGGTGAGCCTTGAA	TCTCCGGGATCGGTCGCGTT
