# Supplementary material for: PELP1 inhibition by SMIP34 reduces endometrial cancer progression via attenuation of ribosomal biogenesis
Source: Mol Oncol. 2023 Nov 1;18(9):2136–56. doi: 10.1002/1878-0261.13539 (PMC11467795; doi:10.1002/1878-0261.13539)
Supplement: Supplementary file 2 — Table S2. Endometrial tumor tissue Demographic Information. [file MOL2-18-2136-s001.pdf]

Endometiral Tumor Tissue Demographic Information					
	**Sample**	**Stage**	**Histology**	**Grade**	**Stains (Pathology)**
1	EEC-3	IA	Endometrioid	Grade 1	MSH2, MSH6, MLH1, PMS2 intact
2	EEC-25	IIIC1	Endometrioid	Grade 2	ER/PR^+
3	EEC-33	IA	Endometrioid	Grade 3	MLH1, MSH2, MSH6, PMS2 intact
4	EEC-34	IB	Endometrioid	Grade 1	intact MSH2, MSH6; MLH1, PMS2 deficient (MLH1 methylated)
5	EEC-36	IA	Endometrioid	Grade 1	intact MLH1, MSH2, MSH6, PMS2; ER/PR^+
6	EEC-41	IIIC2	Endometrioid	Grade 2	MSH2, MSH6, MLH1, PMS2 intact
7	EEC-57	II	Endometrioid	Grade 1	MSH2, MSH6, MLH1, PMS2 intact; ER/PR^+
8	EEC-53	IA	Endometrioid	Grade 3	MSH2, MSH6, MLH1, PMS2 intact, p53 negative
9	EEC-59	IA	Endometrioid	Grade 1	MSH2, MSH6, MLH1, PMS2 intact; ER/PR^+
10	EEC-60	IA	Endometrioid	Grade 1	MSH2, MSH6, MLH1, PMS2 intact; ER/PR^+
11	EEC-62	IA	Mixed endometrioid carcinoma adenocarcinoma	Grade 1	MSH2, MSH6, MLH1, PMS2 intact
12	EEC-63	IA	Endometrioid	Grade 1	MSH2, MSH6, MLH1, PMS2 intact
13	EEC-64	IA	Endometrioid	Grade 1	MSH2, MSH6, intact, Loss of PMS2, MLH1 (hypermethylation present)
14	EEC-65	II	Endometrioid	Grade 3	MLH1, PMS2, MSH2 intact, MSH6 lost, ER/PR^-
15	EEC-69	IB	Endometrioid	Grade 1	MSH2, MSH6, MLH1, PMS2 intact
16	EEC-73	IIIA	Endometrioid	Grade 1	MSH2, MSH6, MLH1, PMS2 intact; ER/PR pos
17	EEC-79	IA	Endometrioid	Grade 1	MSH2, MSH6, MLH1, PMS2 intact
